# Is Conduction System Pacing Going to Be the New Gold Standard for Cardiac Resynchronization Therapy?

**DOI:** 10.3390/jcm13154320

**Published:** 2024-07-24

**Authors:** Michael Derndorfer, Georgios Kollias, Martin Martinek, Helmut Pürerfellner

**Affiliations:** Ordensklinikum Linz Elisabethinen, Fadingerstraße 1, 4020 Linz, Austriamartin.martinek@ordensklinikum.at (M.M.); helmut.puererfellner@ordensklinikum.at (H.P.)

**Keywords:** CSP, LBBAP, HBP, CRT, BiV-CRT, conduction system pacing, HIS bundle pacing, left bundle branch area pacing

## Abstract

The current gold standard in device therapy for advanced heart failure (HF), which has been firmly established in HF management for more than 25 years, is classical biventricular pacing (BiV-CRT). In the last decade, a new pacing modality called conduction system pacing (CSP) has emerged as a variant for advanced cardiac device therapy. It provides pacing with preserved intrinsic cardiac activation by direct stimulation of the specific cardiac conduction system. The term CSP integrates the modalities of HIS bundle pacing (HBP) and left bundle branch area pacing (LBBAP), both of which have provided convincing data in smaller randomized and big non-randomized studies for the prevention of pacemaker-induced cardiomyopathy and for providing effective cardiac resynchronization therapy in patients with classical CRT-indication (primary approach or after failed CRT). Recent American guidelines proposed the term “cardiac physiological pacing” (CPP), which summarizes CSP including left ventricular septal pacing (LVSP), a technical variant of LBBAP together with classical BiV-CRT. The terms HOT-CRT (HIS-optimized CRT) and LOT-CRT (LBBP-optimized CRT) describe hybrid technologies that combine CSP with an additional coronary-sinus electrode, which is sometimes useful in patients with advanced HF and diffuse interventricular conduction delay. If CSP continues providing promising data that can be confirmed in big, randomized trials, it is likely to become the new gold standard for patients with an expected high percentage of pacing (>20%), possibly also for cardiac resynchronization therapy. CSP is a sophisticated new treatment option that has the potential to raise the term “cardiac resynchronization therapy” to a new level. The aim of this review is to provide basic technical, anatomical, and functional knowledge of these new pacemaker techniques in order to facilitate the understanding of the different modalities, as well as to provide an up-to-date overview of the existing randomized and non-randomized evidence, particularly in direct comparison to right ventricular and classical biventricular pacing.

## 1. Introduction

An era is typically associated with a significant event or discovery that profoundly changes the lives or circumstances of many people. In medicine, one such groundbreaking advancement is cardiac resynchronization therapy (CRT), which has revolutionized treatment options in cardiac device therapy. CRT describes a specific form of cardiac pacing that evolved around 1990 and is widely used today. It is essential for the treatment of patients with advanced heart failure (HF) after exhaustion and in addition to optimized neurohumoral medication, when symptomatic impairment of left ventricular function (LVEF) together with a significant widening of the QRS-complex as an expression of electrical interventricular dyssynchrony is still present [1]. Further indications for CRT are pacemaker-induced cardiomyopathies (PICM [2,3,4]), exercise-dependent left bundle branch block (LBBB [5,6]) and the so-called “pace and ablate” concept [7], consisting of a pacemaker implantation combined with an AV-node total ablation, to name just a few examples.

In order to prevent misunderstandings and based on the new HRS guideline on cardiac physiological pacing [8] (summarizing the concept of biventricular CRT as well as conduction system pacing (CSP)), the previous term “CRT” is replaced by the expression “**BiV-CRT**”, to indicate that it is a cardiac resynchronization therapy achieved through the use of a biventricular system with an RV- and coronary sinus (CS) electrode, as opposed to conduction system pacing (CSP), which can also provide cardiac resynchronization.

**Figure 1:**
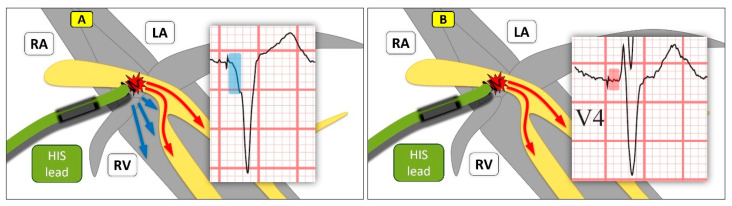
HIS bundle pacing (HBP). (**A**) shows nonselective HBP with both stimulation of the specific conduction system (red arrow) and the adjacent septal myocardium (blue arrows). The resulting QRS-complex is slim but appears somewhat broadened in the beginning by additional myocardial capture (compare preexcitation/pseudo delta wave in WPW, blue segment). (**B**) shows selective HBP, where the electrode is perfectly anchored to the HIS-bundle, exclusively capturing the HIS without excitation of the surrounding tissue. The stimulus then travels over the conduction system until myocardial excitation occurs (intrinsic 35–55 ms; stimulated up to approx. 80 ms), recognizable by a short isoelectric line (red segment) between stimulus and QRS; LA: left atrium; RA: right atrium; RV: right ventricle; HIS: Bundle of HIS; V4: chest lead V4 from a patient’s ECG.

**Figure 2:**
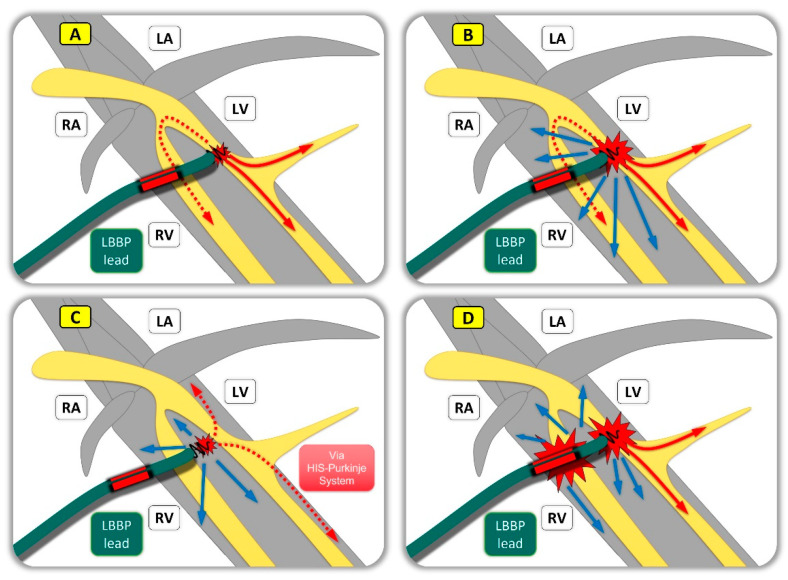
Left bundle branch (area) pacing (LBBAP). Figure 2 shows possible variants of left bundle branch pacing (LBBP), which can usually be differentiated using electrophysiological and ECG criteria. If there is a threshold-dependent change in QRS-morphology from nsLBBP to LVSP, we call it left bundle branch area pacing (LBBAP). Solid red arrows show antegrade capture of the conduction system; blue arrows indicate myocardial capture (**A**) shows selective LBBP/LFP, where the electrode is perfectly anchored to the left bundle branch (or one of its fascicles) exclusively capturing the LBB or left fascicle without excitation of the surrounding tissue (at usually very low output). Excitation therefore initially travels via the conduction system (intrinsic < 35) until myocardial excitation occurs. The isoelectric line, if detectable at all, is much more discrete than during HBP. (**B**) shows nonselective LBBP, where the electrode excites the left bundle branch and the surrounding septal myocardium. (**C**) shows left ventricular septal pacing (LVSP), where the electrode is anchored deep in the interventricular septum but does not directly stimulate the conduction system. Here the stimulation connects to the left subendocardial HIS-Purkinje network (red dotted line) with rapid and very synchronous activation of both ventricles. (**D**) shows anodal nonselective left bundle branch pacing (anodal nsLBBP), with a hybrid activation of the left bundle branch together with anodal ring-capture (through hyperpolarization; bipolar configuration); LA: left atrium; RA: right atrium; RV: right ventricle; LV: left ventricle; LBBP: left bundle branch pacing.

Conduction system pacing by direct stimulation of the cardiac conduction system (and not only the myocardium), is a comparably new technique for cardiac pacemaker therapy. The term itself summarizes the topic of HIS bundle pacing (HBP, Figure 1), which can be divided into a selective and non-selective form (sHBP, nsHBP), as well as the even younger and extensive chapter of left bundle branch area pacing (LBBAP, Figure 2), summarizing the term of left bundle branch pacing (LBBP; capture of the left bundle branch itself), which also provides a selective and non-selective form (sLBBP, nsLBBP), left fascicular pacing (LFP; capture of the left anterior, posterior or septal fascicle more distal to the main bundle branch) and left ventricular septal pacing (LVSP; capture of very fast conducting Purkinje fibers along the left septum without capture of the proximal conduction system), as well as hybrid variants with additional anodal/ring-capture (e.g., anodal nsLBBP). Sometimes several of these forms can be observed in a single patient, depending on the selected output of the device.

Permanent HBP was first published in 2000 [9] as a novel pacing method and gained clinical use around 2010. First, “how-to” articles [10] were published beginning from 2016 in order to make HBP known to a wider range of implanters and to define standardized criteria for the procedures.

LBBP was first presented in 2017 [11] as an alternative to HBP and quickly became widespread among experienced cardiologists as it appears to be an easier technique with shorter procedural time and offers optimal and stable lead parameters. Although QRS duration is often superior, usually with HBP compared to LBBP, HBP has been associated with higher chronic threshold increases, sometimes resulting in a need for lead extraction [12,13,14], which is not a problem for LBBAP [15,16]. HBP is also uniquely associated with atrial oversensing. Building on the knowledge of HBP, innovative and reproducible parameters were established to standardize and facilitate procedures, resulting in the publication of a “Beginner’s Guide to LBBP” [17] in 2019. In the following years, criteria for a successful implantation and parameters to distinguish between different variants of LBBAP were refined and updated steadily. Parallel to these advances in differential diagnostics, the industry acknowledged the increasing need for specialized products and started to adjust to the requirements of implanters. Nowadays, a proper variety of sheaths, pacing-leads, and devices by different companies is available and further innovations are in development.

As the topic of conduction system pacing continues to evolve rapidly and pacemaker centers increasingly recognize the importance of offering CSP in their repertoire, there is a growing need for precise knowledge of specific anatomy and the ability to understand and differentiate the type of CSP achieved. Furthermore, as guidelines struggle to keep up with the fast but dominantly non-randomized scientific advances, an insight of available evidence seems to be crucial to be able to judge what pacing modality might be most beneficial in our patients.

The aim of this review is to provide a basic technical, anatomical, and functional know-how of these new pacemaker techniques in order to simplify the understanding of the different CSP variants. Demonstrative figures are intended to illustrate the principles of HBP and LBBAP and to facilitate the abstract and complex terminology.

A comprehensive literature review on each technology will provide an update on existing randomized and non-randomized evidence, particularly in direct comparison to right ventricular and classical biventricular pacing.

## 2. Comparison of Implant Procedures

BiV-CRTs and CSP systems are typically implanted in operating rooms, electrophysiology labs, or hybrid rooms. According to the hospital’s standards, the procedures are performed in a big variety of settings, ranging from simple local or regional block anesthesia, conscious or deep sedation, up to general anesthesia. After preparation of a subcutaneous or submuscular generator pocket in the left or right infraclavicular region, venous access is achieved by cephalic vein cutdown or by direct puncture of the axillary or subclavian vein using ultrasound or anatomical landmarks. To insert pacemaker electrodes, appropriate sheaths are now inserted using Seldinger’s technique. A reasonable approach would be to start with a right ventricular (RV) lead, which is an essential part of the classic biventricular concept and can serve as a backup lead in CSP. From here on, BiV-CRT and CSP procedures differ from each other:

BiV-CRT requires the fluoroscopic location of the coronary sinus (CS) ostium in order to intubate the CS with specialized wires and sheaths. After contrast application (directly or using a CS balloon) to detect suitable side branches of the great cardiac vein, ideally a posterior vein is selected to advance and fixate a quadripolar electrode in its target position. Standard electrode parameters like sensing, pacing threshold, impedance, and (unwanted) phrenic nerve stimulation (PNS), together with the anatomical and electrical considerations (e.g., inter-electrode distance to the RV-lead), help in deciding whether to finalize the procedure or continue to search for a good CS lead position. The final slithering of the sheath can be challenging in some cases where dislocation of the new lead may occur.

Additional features to aggravate BiV-CRT implantations might be difficulties in finding or passing the CS ostium due to a prominent Eustachian or web-like Thebesian valve, insufficient side branches or target veins for lead insertion, dissection of the CS (intramural or intrapericardial) during sheath manipulation, as well as an inadequate high pacing threshold or phrenic nerve capture in an otherwise suitable position [18,19]. According to landmark studies, the mean procedural duration is between 90 and 180 min (e.g., 2.7 h in MIRACLE [20] or 164 min in COMPANION [21]), but as advances in technology have been made over the years, many clinics nowadays suggest 70 to 120 min for a BiV-CRT intervention.

As CSP implantations are procedures mainly guided by a 12-lead ECG and electrophysiology signals (EP), either a built-in or mobile EP solution has to be available on site. It is needed to record intracardiac signals like HIS or LBB potentials (poHIS, poLBB [22,23,24]), observe changes in injury potentials (as markers for sufficient lead fixation in HBP or parameters for lead depth and immanent perforation in LBBP), perform programmed stimulation in some cases with difficulties to discriminate between CSP and myocardial capture, and to document output-dependent changes in important timings like the so called R-wave-peak-time in lead V6 (V6-RWPT) and the “V6-V1-interpeak interval” that have been published [25,26,27,28] previously. These two intervals facilitate correct positioning of the electrode and help in deciding when to stop further advancement of the electrode and what type of CSP has been achieved. In order to reach anatomical landmarks that are essential for a successful procedure, several pacemaker manufacturers have developed special sheaths with different curvatures and lengths in recent years, which are now in clinical use. A distinction is made between those with fixed curvature and those with variable curvature (steerable sheath). Especially in LBBP, a second curve to the back is essential to adequately guide the lead to the interventricular septum (IVS) and provide sufficient backup for lead fixation [29].

**Figure 3:**
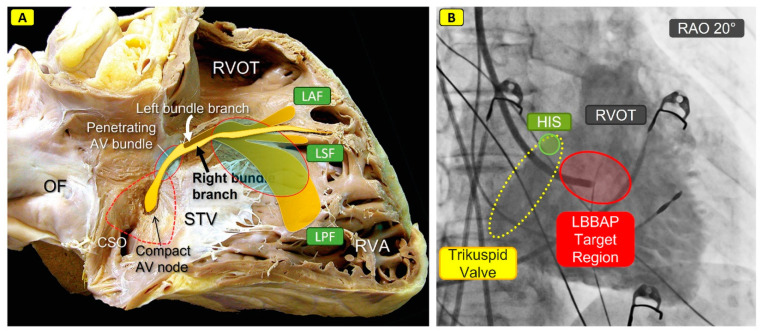
Anatomical and fluoroscopic orientation for CSP procedures. (**A**) shows the positional relationship of the cardiac conduction system in an anatomical specimen (light yellow: right bundle branch, dark yellow/transparent: projection of the left bundle branch on the opposite side of the interventricular septum) and relevant anatomical structures (right atrium/ventricle, RVOT, septal leaflet of the tricuspid valve), as well as the approximate target regions for HBP (small blue shaded oval) and LBBAP (large blue shaded oval). LAF: left anterior fascicle, LSF: left septal fascicle, LPF: left posterior fascicle. Figure 3A is modified from doi: 10.1155/2015/547364. Epub 2015 Nov 19. Copyright © 2015 Damián Sánchez-Quintana et al. (**B**) shows, analogous to (**A**), a fluoroscopic orientation aid in a right anterior oblique 20° projection (RAO) for an approximative estimation of the HIS bundle or the LBBAP target region after contrast administration; OF: foramen ovale; STV: septal leaflet of the tricuspid valve; RVOT: right outflow tract; RVA: right ventricular apex; LAF: left-anterior fascicle; LSF: left-septal fascicle; LPF: left-posterior fascicle; HIS: suggested position of the HIS-bundle; LBBAP: left bundle branch area pacing; CSO: coronary sinus ostium.

The CSP procedure itself is usually first performed under fluoroscopic guidance (Figure 3) to obtain an approximate anatomical orientation; this is followed by EP fine-tuning. For the purpose of HBP, the pacemaker lead is inserted into the right atrium (RA) or proximal right ventricle (RV) near the suggested HIS bundle location using the above-mentioned tools while carefully monitoring the HIS signals. If appropriate signals are detected, the ratio between the size of the atrial (A) and ventricular (V) electrograms can be used to differentiate between proximal and distal HIS positions. Very proximal HIS positions should be avoided to achieve a clear distinction between atrial and ventricular signals and to prevent atrial oversensing and higher chronic thresholds [30]. If the target region seems promising, the pacemaker lead is now anchored into the HIS tissue by applying rotations to the pacing lead until tension starts to build up (usually rotating the entire lead rather than just the anchoring screw). In case of sufficient lead parameters (sensing, pacing threshold, impedance, injury potentials, sHBP, nsHBP), the sheath is then removed in a similar way to BiV-CRT and the procedure is finalized. Possible complications or aggravating factors during HBP include difficulties in finding an appropriate HIS position as the HIS bundle might be located deep inside the muscular part of the interatrial septum or well encased within isolating tissue, insufficient support from sheaths, as well as challenges related to sheath stability. Additionally, low a sensing amplitude and a comparatively high pacing threshold with potentially further increase in postoperative follow-up may occur. Macro- and micro-dislocations (lead position unchanged, but loss of or change in HBP) are possible. Pacing thresholds below 1.5 V@0.5 ms should be targeted, although values up to 2.0–2.5 V@0.5 ms may be accepted in certain cases, which can lead to increased battery consumption, similar to high pacing thresholds in BiV-CRT in some patients. In the case of LBBP, locating the HIS is optional but can be an important landmark for anatomical orientation. Introducing the sheath into the right ventricular outflow tract (RVOT) and recording a contrast bolus for delineation of the tricuspid valve, RV, pulmonary artery, and proximal interventricular septum (IVS) is a convenient option to quickly find the desired target region (Figure 3).

From this point on, the fine adjustment is performed based on ECG parameters by stimulation via the pacemaker lead which has not yet been fully screwed in. After confirming an appropriate initial QRS morphology as described in resent guidelines, the lead is now carefully advanced into the IVS. This process requires a step-by-step approach, especially paying attention to changes in lead impedance, injury potentials, and transitions in the 12-lead ECG morphology together with proper changes in the V6-RWPT and V6/V1-interpeak interval. A continuous connection to the pacing lead might be of advantage in this situation to monitor all those parameters in real time. A poLBB can be observed in many cases and usually helps for further verification of CSP. Confirmation of adequate left bundle branch pacing (LBBP) and its differentiation from various forms of LBBAP may require some experience and is described in detail in a recently published European consensus paper [31]. LBBP typically exhibits an excellent acute and chronic pacing threshold (e.g., 0.7 V@0.4 ms), similar to a conventional RV lead. During lead-implantation for LBBAP, it is important to maintain sufficient distance from the tricuspid valve apparatus to avoid septal leaflet restrictions. Possible factors complicating the procedure include entanglement (getting stuck to the septal tricuspid leaflet or fibrous tissue), the drill effect (temporary creation of a drill channel that prevents secure lead anchoring at that specific location), and the screwdriver effect (fast lead progression during rotations with the risk of perforation). In the rare event of such a perforation through the IVS into the left ventricle, the lead must be withdrawn and repositioned. Rare reports on isolated cases of lead fracture [32,33,34], septal coronary vein fistulas [35], septal coronary artery fistulas, [36] and septal hematoma [37] (with spontaneous resolution) are described.

## 3. Evidence on BiV-CRT

As BiV-CRT has been clinically available for more than 25 years, it can rely on a solid scientific background of multiple randomized studies:

The first patent for this novel therapy approach was granted in 1990. Reports on favorable hemodynamic response with encouraging clinical follow-up in a patient with heart failure and left bundle branch block (LBBB) were published in 1994 [38]. Until then, epicardial lead placement was the procedural standard. An important step in the further development of BiV-CRT therapy was the first description [39] of a transvenous approach by positioning the lead over the coronary sinus (CS). Small, randomized studies were published in 2001 (MUSTIC [40], PATH-HF [41]) and were able demonstrate an improvement in the quality of life (QoL) of patients as well as a so-called “reversed remodeling” of the left ventricle (LV), which describes a reversal of the adverse cardiac remodeling processes observed in heart failure. A breakthrough was finally achieved in the following year, where a large-scale double-blind randomized study (MIRACLE [20], 453 patients, BiV-CRT “on” or “off”) was able to demonstrate an improvement in walking distance, QoL, exercise tolerance, NYHA stage, and LV reversed remodeling within 6 months of follow-up in the BiV-CRT group, compared to patients in the control arm receiving only a defibrillator. COMPANION [21] was the first study to compare optimized heart failure therapy (including diuretics, ACE inhibitors, beta blockers, and spironolactone) with either a BiV-CRT pacemaker (CRT-P) or BiV-CRT defibrillator (CRT-D); for the first time, a hard clinical endpoint with a 20% reduction of the combined endpoint (death or hospitalization of any cause) was achieved. However, the mortality reduction was only significant in patients with activated defibrillator therapy (ICD) and NYHA class I to III. In 2005, and with an average follow up of 29.4 months, the CARE-HF [42] trial proved a significant mortality benefit from BiV-CRT-P on top vs. optimized heart failure medication alone along with clinical and echocardiographic improvements.

Following these convincing results, BiV-CRT therapy was established as an important adjunctive therapy for symptomatic HF patients (NYHA class II and IV) with reduced LVEF and a wide QRS complex. Between 2008 and 2010, additional landmark studies, such as RAFT [43] (1798 patients, NYHA class II and III), REVERSE [44] (610 patients, NYHA class I and II), or MADIT-CRT [45] (1820 patients, NYHA class I and II), demonstrated improvement in LVEF and a reduction in heart failure-related hospitalization in less symptomatic heart failure patients. However, they did not achieve an independent mortality benefit, possibly due to the low overall mortality in this NYHA class I/II group.

In patients with normal LVEF, the clinical effects of BiV-CRT vs. RVP were examined in two studies (50 patients and 149 patients [46]) and showed preservation of the left ventricular function and lack of adverse remodeling in long-term FU with resynchronization therapy. In the PREVENT-HF [47] study, 108 patients with preserved LVEF and an anticipated high pacing percentage > 80% were randomized to BiV-CRT or apical RV pacing. BiV-CRT therapy was neutral here and could not show any advantage.

The importance of QRS duration regarding the indication for BiV-CRT in patients with heart failure and reduced ejection fraction (HFrEF) must be explicitly emphasized, since CRT has the ability to widen an existing narrow QRS-complex by creating artificial dyssynchrony [48]: In 2013, two studies were published (LESSER-EARTH [49], ECHO-CRT [50]; symptomatic HF, LVEF ≤ 35%, QRS ≤ 120 or 130 ms) in which the indications were derived from mechanical dyssynchrony (assessed by echo parameters) rather than electrical dyssynchrony (indicated by a (left) bundle branch block). Both studies had to be stopped early due to clinical deterioration and risk to the patients (significant decline in the 6-min walk test, non-significant trend of increased HF-associated hospitalization; 81% higher mortality in the “CRT on” group (*p* = 0.02, HR 1.81)).

Overall, the evidence for BiV-CRT therapy is supported by a large number of solid and large randomized studies, so that the current HF guidelines [51,52] of the cardiology societies can base their recommendations on a large amount of well-founded data. The best evidence for BiV-CRT is in patients with symptomatic HF together with a LVEF < 35%, LBBB > 150 ms, and already optimized HF medication (Class I, LOE: A).

For non-LBBB patients, i.e., those with right bundle branch block (RBBB), but also LBBB-like block (who do not meet strict LBBB criteria) and diffuse interventricular conduction delay (IVCD), there is no solid evidence of a reduction in death and/or HF hospitalization through a BiV-CRT system to date (large meta-analyses [53]), so that recommendation levels here have been downgraded in recent pacemaker guidelines (class IIa and IIb) [54].

## 4. Evidence on CSP

Comparable to intrinsic LBBB, right ventricular pacing (RVP), can lead to the development of pacemaker-induced cardiomyopathy (PICM) via electrical and consequently mechanical cardiac dyssynchrony. A large-scaled meta-analysis [55] of corresponding studies including around 58,000 patients showed a pooled prevalence of 12% (95% confidence interval: 11–14%). The most important risk factors were the preexisting LV function at the time of pacemaker implantation, intrinsic QRS width, the proportion of RV pacing, and the width of the paced QRS complex. In patients with a pacing proportion ≥ 40%, multiple studies showed an incidence between 5.9–39% (FU 0.7–16 years).

Therefore, the first CSP studies were performed in direct comparison with RV pacing and are now being reflected in a recent meta-analysis [56] of 13 studies (2348 patients; normal or slightly reduced LVEF), where HBP (vs. RVP) was associated with a narrower QRS complex and a significantly improved LVEF (at FU > 1 year: *p* < 0.001) in connection with a significantly lower risk of HF-related hospitalization (*p* = 0.03) and a trend towards a reduced overall mortality (*p* = 0.12). A possible disadvantage of HBP compared to RVP seems to be a higher risk for the need of lead revisions (5–7% [57]), as noted in many case-series. Observed reasons were a high HIS pacing threshold, lead dislocations, and a loss of capture or incorrect sensing on the pacing lead. Therefore, in some cases of HBP (e.g., pacemaker dependence like in AV node total ablation or complete AV block), additional implantation of an RV backup electrode is recommended. The most noticeable advantages of HBP are its characteristic as the only method so far providing truly physiological activation of both ventricles as well as its anatomical aspect that does not require lead placement via the tricuspid valve in most cases, therefore avoiding pacemaker induced tricuspid regurgitation.

To compare LBBAP vs. RVP, a large multicenter registry study [58] with 703 patients (LBBAP 321 patients, RVP 382 patients) was published in 2021. During a long FU of 3 years, LBBAP demonstrated a significantly better performance concerning the composite primary endpoint (mortality, heart failure related hospitalization, or need for a BiV-CRT upgrade (10% event rate for LBBAP; 23.3% for RVP; HR 0.46; *p* < 0.001)), especially in patients with a higher pacing proportion > 20% (8.4% event rate for LBBAP; 26.1% for RVP; HR 0.32; *p* < 0.001). LBBAP was also associated with a significant reduction in mortality (7.8% for LBBAP; 15% for RVP; HR 0.59; *p* = 0.03) and HF-related hospitalization in this clientele (LBBAP: 3.7%; RVP: 10.5%; HR 0.38; *p* = 0.004). The same was true in another prospective study [59] published in 2021, where 386 consecutive patients with AV block, preserved LVEF, as well as a high percentage of pacing (~85%) received either LBBAP or RVP and were followed for 11.4 ± 2.7 months. Patients with LBBAP preserved LV function better (LVEF of 62.6% vs. 57.8%) and had a significantly lower incidence of HF-related hospitalization or the need for upgrade to a BiV-CRT system (LBBAP: 2.6%; RVP 10.8%; *p* < 0.001%).

Recently, a systematic literature review and meta-analysis [60] of six articles including 144 patients was performed on HIS-Purkinje conduction-system pacing (HPCSP) for pacing-induced cardiomyopathy, aiming to assess the efficacy and clinical benefit of upgrading to HPCSP in patients with PICM after chronic right ventricular pacing. The authors were able to show a significant mean increase in QRS-duration (QRSd) during RVP (127 ± 29 ms at baseline to 175 ± 19 ms; *p* < 0.001) and significant narrowing after HPCSP (116 ± 18 ms; *p* < 0.001). During a mean follow-up of 17.9 ± 10.5 months, LVEF significantly improved after upgrading to HPCSP (from 35 ± 8% at baseline to 48 ± 12%; *p* < 0.001), therefore confirming feasibility and improvement in electrical synchrony and cardiac function with CSP. These results are consistent with a 2016 study [61] that aimed to calculate the incidence of RV-PICM and identify predictors in patients with complete heart block and preserved LVEF. RVP in >20% of time was associated with a higher risk of PICM; BiV-CRT response was high in this patient group as well.

Another meta-analysis [62] from 2023 (25 trials; 4250 patients), summing up data on LBBP vs. conventional RVP in patients with bradycardia and conduction system disorders, was able to prove a shorter QRSd in the LBBP group (*p* < 0.001), both at implantation and during follow up, as well as a significant reduction in LVEF (*p* < 0.001) in the RVP-group. LBBP achieved similar pacing thresholds (*p* = 0.86) and higher R wave amplitudes (*p* < 0.05) than RVP, while lead-related complications had no difference between the two groups (LBBP = 1.90% vs. RVP = 1.72%; *p* = 0.71). Therefore, the authors concluded that LBBP maintains electrical and mechanical synchrony of the ventricles, shows a reduction in heart-failure hospitalization compared to RVP, and has excellent pacing parameters without compromising safety.

Following these encouraging data for CSP in patients with structurally healthy hearts, the first implantations were performed in patients with impaired left ventricular function and BiV-CRT indication who either had a history of failed BiV-CRT procedure (lead failure, e.g., unsuitable CS veins or phrenic nerve stimulation) or who were so-called “CRT non-responders”, i.e., patients in whom BiV-CRT therapy did not achieve clinical improvement. In a multicenter study (16 hospitals), a total of 200 patients were treated with LBBAP (approx. 75% lead failure group). The mean LBBAP stimulus thresholds were excellent (0.68 ± 0.35 V@0.40 ms), as was the sensing at implantation (10.4 ± 5 mV), and parameters remained stable in the FU over 12 ± 10.1 months. LBBAP caused a significant QRS narrowing from 170 ± 28 ms at baseline to 139 ± 25 ms (*p* < 0.001), resulting in an improvement in LVEF from an initial 29 ± 10% before implantation to 40 ± 12% (*p* < 0.001) during FU. In patients with previous technical problems during BiV-CRT, LBBAP had a lower mortality risk and fewer HF hospitalizations compared to known clinical non-responders to BiV-CRT.

## 5. Direct Comparison of CSP vs. BiV-CRT

**Figure 4:**
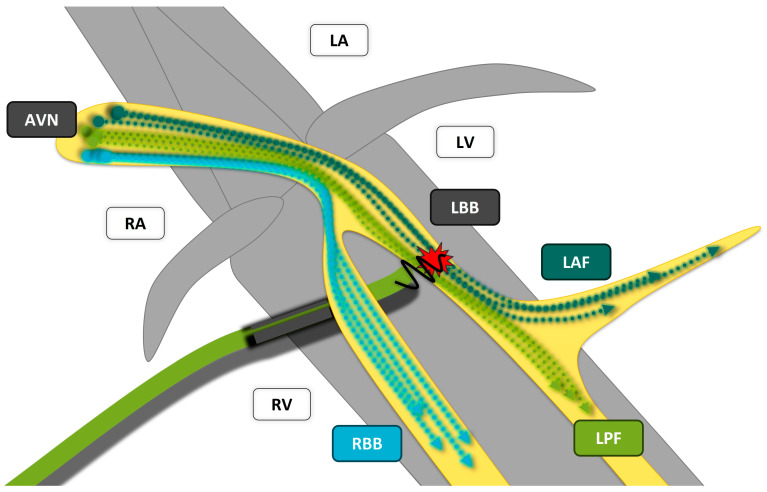
Principle of longitudinal dissociation of the cardiac conduction system, which assumes that the conduction fibrils already have a fixed arrangement proximally in the HIS bundle and are thus predetermined to form the left or right fascicle. Accordingly, it should be possible in many cases to correct a bundle branch block already proximally at the HIS bundle or left bundle branch; AVN: AV node; RA: right atrium; LA: left atrium; RV: right ventricle; LV: left ventricle; LBB: left bundle branch; dark green arrows: fibers forming the left anterior fascicle (LAF); light green arrows: fibers forming the left posterior fascicle (LPF); light blue arrows: fibers forming the right bundle branch (RBB).

Today, there is consensus that resynchronization of both ventricles by correcting an existing left bundle branch block is essential for a successful device therapy in heart failure. BiV-CRT achieves a coordinated contraction by creating two wave fronts, one via the RV electrode (or intrinsic conduction via the right bundle branch), the other via the CS electrode located epicardially in a left-posterior side branch of the great cardiac vein. To understand whether CSP has the potential to correct a bundle branch block, it is worth taking a look at a histopathological publication [63] from 2016: The microstructure of the cardiac conduction system shows a so-called “longitudinal dissociation” (Figure 4), a term which describes that the conduction fibrils already have a fixed arrangement proximally in the HIS bundle and are thus predetermined to form the left or right fascicle. This provides an explanation for why a LBBB occurs proximal in the HIS bundle in 46% of cases and in the proximal left bundle branch in 18% of cases [64], and can be corrected at the HIS or left bundle branch if the fibers distal to the disorder can be re-recruited.

Based on this knowledge, large multicenter registries began to compare CSP directly with BiV-CRT. Randomized observations on these new technologies are currently limited to very small series: in a randomized crossover study [65] (29 patients), HBP was able to cause a significant narrowing of the QRS together with a response equivalent to that of BiV-CRT over 6 months. The HIS alternative [66] study randomized 50 patients to HBP vs. BiV-CRT, with 72% successful correction of the LBBB in the HBP group and comparable improvement in LVEF in both groups (increase of 16 ± 7% (HBP) and 13 ± 6% (BiV-CRT)) in the intention-to-treat analysis (ITT, i.e., including patients in whom the bundle branch block could not be corrected with HBP). The stimulation thresholds for HBP were significantly higher at implantation and at FU at 6 months (2.3 ± 1.4 V vs. 1.4 ± 0.5 V; *p* < 0.01). In the per-protocol analysis (i.e., only those patients in whom the LBBB was successfully corrected using HBP), HBP achieved a significantly higher improvement in LVEF compared to BiV-CRT in the case of successful LBBB correction (LVEF in FU: 48 ± 8% vs. 42 ± 8%; *p* < 0.05).

LBBAP in a patient population with CRT indication was presented for the first time in 2021 as part of a multicenter registry [67] (325 patients), where excellent and stable electrode parameters and a favorable clinical outcome were recorded at an FU of 6 ± 5 months (significant improvement of QRS duration, NYHA stage, LVEF, and LVEDD; even greater advantage with true LBBB compared to non-LBBB). The first randomized data on LBBAP vs. BiV-CRT were derived from the 2022 LBBP-RESYNC [68] pilot study (40 patients, symptomatic non-ischemic cardiomyopathy; LVEF 29.7 ± 5.6%; LBBB), where LBBAP was successful in 85% of the cases and the ITT analysis after 6 months showed a significantly greater improvement in LVEF (difference +5.6%; *p* = 0.039) and greater reduction in NT-proBNP (−1071.80 pg/mL) and was able to demonstrate comparable clinical endpoints (e.g., NYHA, 6-min walking test) in the LBBAP group.

The year 2023 revealed the most comprehensive data on the direct comparison of the previous gold standard BiV-CRT vs. CSP [69] (477 patients with LVEF 26 ± 6% and class I or II indication for CRT, FU 27 ± 12 months) and BiV-CRT vs. LBBAP [70] so far (1778 patients, 69 ± 12 years, observational, consecutive, LVEF 27 ± 6%, and class I or II indication for CRT, FU 33 ± 16 months). In the former study, 219 patients with BiV-CRT and 258 patients with CSP (HBP: 87, LBBAP: 171) were compared. CSP was able to achieve a significantly narrower QRS complex (CSP: 133 ± 21 ms vs. BiV-CRT: 153 ± 24 ms; *p* < 0.001), a significant and superior increase in LVEF (CSP: 39, 7 ± 13% vs. BiV-CRT: 33.1 ± 12%; *p* < 0.001), and a significantly lower primary outcome (death or HF-related hospitalization; CSP: 28.3% vs. BiV-CRT: 38.4%; HR 1.52; *p* = 0.013). The second multicenter study included 981 patients in the BiV-CRT arm and 797 patients in the LBBAP arm. LBBAP was able to significantly narrow the QRS in this patient population (QRS under LBBAP 128 ± 19 ms vs. 161 ± 28 ms at baseline, *p* < 0.001) and achieve a significantly narrower QRS complex compared to BiV-CRT (128 ± 19 ms vs. 144 ± 23 ms, *p* < 0.001). There was a significant improvement in LVEF with both LBBAP and BiV-CRT (LBBAP at baseline 27 ± 6% vs. 41 ± 13% in FU, *p* < 0.001; BiV-CRT at baseline 27 ± 7% vs. 37 ± 12% in FU, *p* < 0.001); the increase in LVEF was again significantly better with LBBAP than with the gold standard BiV-CRT (increase of 13 ± 12% vs. 10 ± 12%, *p* < 0.001). The primary outcome (composite of death or HF-related hospitalization) in the multivariate regression analysis was again significantly better with LBBAP compared with BiV-CRT (20.8% vs. 28%; HR 1.495; *p* < 0.001). Overall, it was concluded that in this non-randomized multicenter study including 1778 consecutive patients with heart failure and CRT indication over a follow-up of 33 ± 16 months, LBBAP had an improved outcome compared to BiV-CRT and could represent a reasonable therapeutic alternative.

Also in 2023, the multicentric I-CLAS [71] study compared the incidence of VT/VF and new onset AF among patients with no history of AF. Included were patients with a left ventricular ejection fraction ≤ 35% who underwent BVP or LBBAP for CRT. As LBBAP was associated with a lower incidence of sustained VT/VF and new onset AF compared with BVP. The conclusion drawn from this trial was that physiological resynchronization by LBBAP may be beneficial for patients, compared with BiV-CRT. Finally, a meta-analysis [72] of available studies on BiV-CRT vs. LBBAP, published in 2024, should be mentioned: it summarizes 13 studies (3239 patients, 12 observational trials, 1 randomized trial) comparing LBBAP and BiV-CRT for cardiac resynchronization in patients with HFrEF. LBBAP was associated with a significant absolute risk reduction of 3.2% in all-cause mortality (9.3% vs. 12.5%, RR 0.7, *p* = 0.001) as well as an 8.2% reduction in HF-related hospitalization (11.3% vs. 19.5%, RR 0.6, *p* < 0.00001) when compared to BIVP. LBBAP also resulted in reductions in procedural time (minus 23.2 min, *p* = 0.02) and fluoroscopy time (minus 8.6 min, *p* < 0.001), as well as a significant reduction in QRS duration (minus 25.3 ms, *p* < 0.00001) and a greater improvement in LVEF of 5.1% (*p* < 0.001) vs. BiV-CRT.

One of the most recent studies available on LBBAP so far studied sex differences [73] in LBBAP vs. BiV-CRT, as we know from previous studies that women respond more favorably to BiV-CRT than men. A total of 539 patients (men: 376, women: 163) were included in this multicenter prospective registry. The authors concluded that there was no significant difference in the composite primary outcome (HF-related hospitalization and all-cause mortality) when comparing men and women receiving LBBAP (*p* = 0.46), but as men tend to benefit less from BiV-CRT than women, men undergoing LBBAP showed a lower risk of HF-related hospitalization and all-cause mortality compared to those who received standard BiV-CRT (LBBAP: 29.9%, BiV-CRT: 46.5%, *p* = 0.004). In women, no statistically significant difference concerning the primary endpoint could be observed (LBBAP: 24.14% vs. BiV-CRT: 36.2%; *p* = 0.23).

Studies from Section 6 comparing CSP vs. BiV-CRT are summarized in Table 1.

## 6. Future of Role of Conduction System Pacing

**Figure 5:**
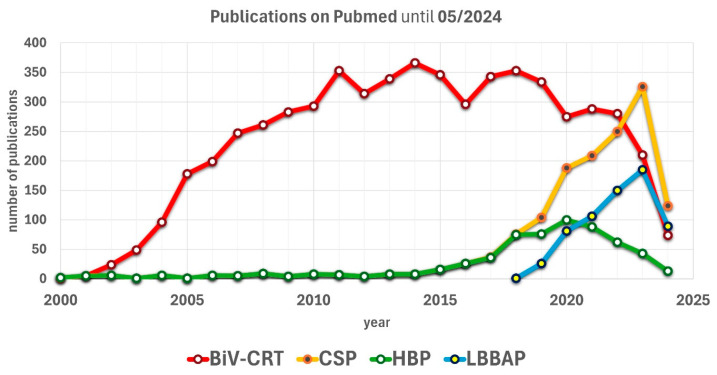
PubMed-listed publications on the topics of BiV-CRT or CSP over time. Figure 5 shows the combined PubMed-listed publications on the topics of BiV-CRT vs. CSP and the rising scientific interest in the new variants of physiological pacing, winning the publication race in 2023 for the first time. BiV-CRT: classical biventricular cardiac resynchronization therapy; CSP: conduction system pacing; HBP: HIS bundle pacing; LBBAP: left bundle branch area pacing.

A closer look at the available publications on BiV-CRT, HBP, and LBBAP reveals the extensive data on BiV-CRT, as well as the rapidly increasing interest in these promising new forms of CSP (Figure 5). In the year 2023, CSP managed to win the publication race against BiV-CRT for the first time. This is primarily due to a rapidly growing interest in LBBAP, while publications on HBP are currently showing a declining trend since 2020.

The answer to the question of whether CSP will end the golden era of BiV-CRT depends on how one interprets the term “era” itself; when BiV-CRT was emerging 25 years ago, it ended decades of exclusive RV stimulation. BiV-CRT was in vogue at this time, was being intensively researched, continuously provided promising data, and significantly changed the treatment of heart failure. Today, due to its comprehensive data, BiV-CRT is well anchored in guidelines, a real gold standard and an indispensable part of a guideline-compliant therapy. Its area of application is also well defined: we know when we should use BiV-CRT (e.g., symptomatic HF, LVEF ≤ 35%, LBBB) and when not to (e.g., QRS < 130 ms in HF).

A similar development can be observed for conduction system pacing: the entirely new therapeutic approach of maintaining the intrinsic/physiological excitation of the heart has opened up a completely new field of research that currently offers fertile ground for many researchers. CSP has furthermore initialized a discussion on the legitimation of RV pacing in many cases; nowadays, based on the available data, we have to seriously consider whether it is still justified to place a standard RV lead in patients with an expected high pacing ratio of >20% (e.g., any AV block, pace and ablate) and an acceptable life expectancy, knowing that a substantial proportion of them will develop a pacemaker-induced CMP, will have a higher risk of HF-related hospitalization, and—most importantly—have a higher mortality rate. Rather than creating a wide LBBB with accompanying dyssynchrony through RV pacing, the primary goal should be to preserve the patient’s own physiological ventricular activation (which should be assumed as the true gold standard) as well as possible. Some might claim that the majority of patients still do not develop PICM, that RV pacing is simpler and cheaper, and that a PICM can be easily remedied by upgrading to BiV-CRT or CSP, therefore opening up a fundamental discussion depending on many factors, such as personal experience or the technical or financial possibilities. Another advantage of CSP is the fact that it generates an excellent (in HBP even perfectly physiological) QRS, which facilitates the programming of devices in AVB I° and II°, since excessively long AV intervals (due to company-specific mechanisms, e.g., MVP, VIP, Rhythmiq, IRS plus) no longer have to be accepted for avoidance of potentially harmful RV pacing. In these cases, CSP enables the programming of meaningful AV intervals while accepting a higher proportion of (physiological) pacing.

Concerning cardiac resynchronization, CSP nowadays provides us with elegant therapy alternatives after failed BiV-CRT, either in the case of lead failure or in clinical BiV-CRT non-responders. Pace and ablate strategies could become the domain of CSP, as many of these patients suffer from treatment-resistant AF, but still have a narrow QRS complex that needs to be preserved as well as possible. In particular, LBBAP will play an increasingly important role here, as it provides excellent sensing and low and stable stimulus thresholds and is located far from Koch’s triangle, and therefore not in the danger zone of AV node total ablation. In addition, the implantation of just a single LBBAP electrode connected to a single-chamber pacemaker reduces the risk of venous complications and offers a high-end therapy (full cardiac resynchronization) at low cost in patients with permanent atrial fibrillation. Finally, recently published large, non-randomized multicenter studies (mentioned above) have not only demonstrated that CSP is equal to BiV-CRT in terms of clinical parameters and outcomes, but have also hypothesized that CSP may potentially provide additional and significant improvement for the patient. If large, randomized studies are able to confirm these assumptions, then CSP will be represented in future devices and heart failure guidelines with good evidence and a high level of recommendation. Many implanting centers will then have no alternative to offering their patients the various options of CSP.

**Figure 6:**
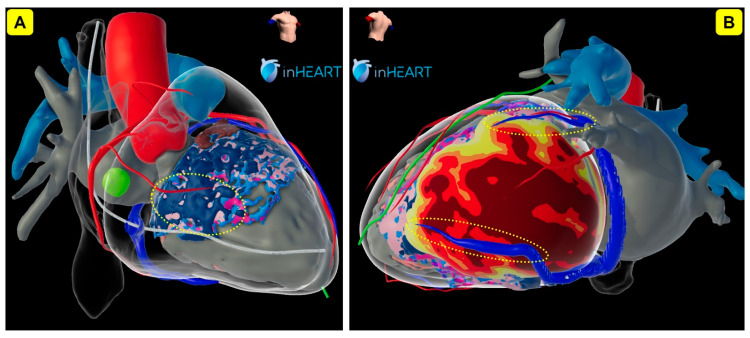
Visualization of CSP—and BiV-CRT—considerations using cardiac 3D reconstructions. (**A**) RAO view of a patient’s heart, showing a vast area of scar (light blue) and dense scar (dark blue) in the mid-myocardial and subendocardial tissue (pink/light pink) of the interventricular septum, possibly making this patient unsuitable for a pacing lead in the LBBAP region (yellow dotted area) (**B**) Left (postero-)lateral view of a patient’s heart, showing a big posterolateral scar with extensive wall thinning (dark red: 1 mm, red: 2 mm, orange: 3 mm, yellow: 4 mm of wall thickness) after a previous myocardial infarction, possibly making the posterolateral veins (yellow dotted areas) unsuitable for a standard BiV-CRT system. CSP: conduction system pacing; LBBAP: left bundle branch area pacing; BiV-CRT: classical biventricular cardiac resynchronisation therapy; RAO: right anterior oblique.

The recent HRS guidelines [1] have already taken CSP to the next level as they proposed the term “cardiac physiological pacing” (CCP) for HBP, LBBAP, and BiV-CRT, therefore allowing the implantation of a CSP lead instead of a coronary sinus electrode in many indications. In terms of not being the main focus of interest anymore and being pushed out of the spotlight by the new therapeutic options of CSP, it currently seems as if the era of classical BiV-CRT might begin to fade. However, BiV-CRT is unlikely to disappear from the market in the near future because it is a solid and well-established therapy with many implanters around the world who were trained for their whole career and are highly skilled in CS lead implantation. Also, intensive industry research and ongoing optimizations over recent decades were able to transform BiV-CRT systems into sophisticated and extensive therapy options for a vast range of different situations. We will definitely continue to require BiV-CRT for patients with diffuse conduction disorder where proximal stimulation of the conduction system might fail to resolve a distal conduction block. However, on the other hand, we will be in need of new technologies like CSP when we have to treat HF patients with (postero-)lateral LV scars, where a standard coronary sinus electrode might not be a sufficient option. Imaging (e.g., Echo, MRI) will possibly play an increasingly important role in decision making (Figure 6). In individual patients with severely dilated hearts, we will sometimes have to use hybrid technologies (e.g., BiV-CRT + CSP, so-called “HOT-CRT” or “LOT-CRT” systems) and we will continue using BiV-CRT devices in CSP due to their multiple connection options, although specialized CSP devices would be preferred.

At the moment, we are observing a transition phase, where more and more new centers are starting to implement CSP and finding their way to have a perfect and functional setup (e.g., EP Lab, mobile solutions, 3D mapping). Still, it might take some time until CSP is going to be routinely available in the majority of hospitals and will be able to fully cover the increasing need for high-quality pacemaker therapies. However, guidelines and recommendations from cardiac societies can accelerate such developments. Replacing all pacemakers with CSP (e.g., including sick sinus indications and shorter life expectancy) does not always seem to be realistic or clinically useful.

CSP is already starting to reshape the device market and bring a breath of fresh air with numerous new possibilities in advanced patient care. So far, we have only seen the first steps of CSP and can expect gradual optimizations and interesting further developments, some of which have already been announced by companies. Only the future can tell whether CSP will one day have shaped an era of CSP to the same extent as BiV-CRT did, or—due to rapid changes in technology—whether several innovations will have to share an era together (e.g., CSP and leadless pacemakers). It is plausible that there will also be a peaceful coexistence of conventional and modern forms of device therapy, which will offer a large degree of freedom in surgical modalities for implanters. Since it currently seems that the miniaturization of pacemakers (leadless pacing) is also a trend with great future potential, it is to be expected that there will also be hybrid solutions here together with CSP.

Concerning future developments in cardiac pacing, perhaps a quote from Mark Twain might be most suitable to answer the initial question of whether conduction system pacing is going to be the new gold standard for cardiac resynchronization therapy: “Forecasts are difficult, especially when they concern the future”.

## Figures and Tables

**Table 1:** CSP vs. BiV-CRT. This table summarizes studies that directly compare CSP (including HBP, LBBAP, or both) vs. BiV-CRT. It provides a quick overview of study design, patient allocation, mean follow-up duration, and relevant outcomes.

Study	Study Design	Patients‘ Allocation	Mean FU	Outcomes
Lustgarten et al. Heart Rhythm 2015 [65]	Randomize crossover multicenter	HBP: 29 BiV-CRT: 29 pts	12 month	HBP and BiV-CRT both improved LVEF, NYHA class, 6MWT and QoL significantly
“HIS-Alternative” Vinther et al. JACC EP 2021 [66]	Randomized prospective single-center	HBP: 25 pts BiV-CRT: 25 pts	6 month	LVEF significantly higher and LVESV significantly lower in HBP group at 6 months
Vijayaraman et al. JACC EP 2021 [67]	Observational retrospective multicenter	LBBAP: 325 pts	6 ± 5 month	QRS narrowing; LVEF and NYHA class improvement
“LBBP-RESYNC” Wang Y et al. JACC EP 2022 [68]	Randomized prospective multicentre	LBBAP: 20 pts BiV-CRT: 20 pts	6 month	Greater LVEF improvement and higher reduction in LVESV and NT-proBNP with LBBAP
Vijayaraman et al. Heart Rhythm 2022 [69]	Observational retrospective multicenter	HBP: 87 pts LBBAP: 171 pts BiV-CRT: 219 pts	27 ± 12 month	Superior increase in LVEF with CSP; significantly lower primary outcome (death or HF-related hospitalization with CSP vs. BiV-CRT
Vijayaraman et al. JACC 2023 [70]	Observational retrospective multicenter	LBBAP: 797 pts BiV-CRT: 981 pts	33 ± 16 month	Significant improvement in LVEF with both LBBAP and BiV-CRT but significantly better with LBBAP; Primary outcome (composite of death or HF-related hospitalization) significantly better with LBBAP
“I-CLAS” Herweg et al. Circulation 2024 [71]	Observational retrospective multicenter	LBBAP: 797 pts BiV-CRT: 981 pts	25.2 ± 15 month	Occurrence of VT/VF, VT-storm, new-onset AF > 30 s and AF lasting > 24 h was significantly lower with LBBAP compared with BiV-CRT
Diaz et al. JICE, 2024 [72]	Meta-analysis	LBBAP: 1338 pts BiV-CRT: 1901 pts	25.8 month	Significant reduction in all cause mortality, HF-related hospitalization when compared to BiV-CRT
Tedrow et al. JACC 2024 [73]	Observational prospective multicenter	women: LBBAP: 58 pts women: BiV-CRT: 105 pts men: LBBAP: 127 pts men: BiV-CRT: 249 pts	400.5 days	No sex differences in patients undergoing LBBAP for CRT; Lower risk of HF-hospitalization and all-cause mortality with LBBAP vs. BiV-CRT in men; No difference between LBBAP and BiV-CRT in women

HBP: HIS bundle pacing; BiV-CRT: classical biventricular cardiac resynchronization therapy; LVEF: left ventricular ejection fraction; NYHA: New York heart failure association; 6MWT: 6-min walk test; LBBAP: left bundle branch area pacing; LVESV: left ventricular end-systolic volume); CSP: conduction system pacing; VT: ventricular tachycardia; VF: ventricular fibrillation; QoL: quality of life; AF: atrial fibrillation; HF: heart failure.

## Data Availability

The data presented in this review are available in the corresponding references.

## Abbreviations

**RVP**: right ventricular pacing, **CSP**: conduction system pacing, **HBP**: HIS bundle pacing, **sHBP**: selective HIS bundle pacing, **nsHBP**: non-selective HIS bundle pacing, **LBBP**: left bundle branch pacing, **sLBBP**: selective left bundle branch pacing, **nsLBBP**: non-selective left bundle branch pacing, **LBBAP**: left bundle branch (area) pacing, **LFP:** left fascicular pacing, **LAF**: left-anterior fascicle, **LSF**: left-septal fascicle, **LPF:** left-posterior fascicle, **RBBP**: right bundle branch pacing, **LVSP**: left ventricular septal pacing, **DSP**: deep septal pacing, **BiV-CRT**: classical biventricular cardiac resynchronization therapy, **HOT-CRT**: HIS-optimized CRT, **LOT-CRT**: LBBP-optimized CRT, **CPP**: cardiac physiological pacing, **RVOT**: right-ventricular outflow tract, **CSO**: coronary-sinus-ostium, **OF**: foramen ovale, **STV**: septal leaflet of the tricuspid valve, **IVS**: interventricular septum, **IVCD**: interventricular conduction delay.
